# Expression of transforming growth factor-beta-1 and p27^Kip1 ^in pancreatic adenocarcinomas: relation with cell-cycle-associated proteins and clinicopathologic characteristics

**DOI:** 10.1186/1471-2407-5-98

**Published:** 2005-08-08

**Authors:** Nil Culhaci, Ozgul Sagol, Sedat Karademir, Huseyin Astarcioglu, Ibrahim Astarcioglu, Mujde Soyturk, Ilhan Oztop, Funda Obuz

**Affiliations:** 1 Department Of Pathology, Adnan Menderes University, Faculty of Medicine, Aydin, Turkey; 2 Hepatobiliary Study Group, Dokuz Eylül University, Faculty of Medicine, Izmir, Turkey

## Abstract

**Background:**

The purpose of our study was to investigate the immunohistochemical expression of TGF-β1 and p27 in pancreatic adenocarcinomas and to compare the findings with the clinicopathological features and survival. We also aimed to evaluate the expression of TGF-β1 and p27 in the context of other cell cycle and proliferation markers such as cyclin D1 and Ki-67.

**Methods:**

We examined TGF-β1 and p27 expression immunohistochemically in 63 cases of invasive ductal adenocarcinoma of the pancreas. Standard streptavidin-biotin immunperoxidase method was used for immunostaining and the stained slides were examined microscopically using semiquantitative criteria.

**Results:**

TGF-β1 stained the cytoplasms of the tumor cells in 43 cases [68.3%]. There was a statistically significant difference among TGF-β1 staining scores in terms of clinicopathologic factors such as blood vessel invasion, stage and distant metastasis [p < 0.05]. Of the 63 tumors evaluated 23 [36.5%] were positive for p27 within the nucleus. An inverse correlation was found between p27 immunoreactivity and grade [p < 0.05]. But no significant correlation was found between p27 and other parameters. Among the patients with survival data 27 patients had RO resections and these cases were considered in survival analysis. In the univariate analysis, neither TGF-β1 nor p27 expression was related with patient survival.

**Conclusion:**

Our findings suggest that in pancreatic carcinoma, TGF-β1 expression is related to tumor growth and metastasis. But it is not associated with cell cycle proteins. p27 expression is reduced in pancreatic adenocarcinomas and decreased protein levels of p27 may play a role in the differentiation of pancreatic cancer.

## Background

Pancreatic cancer is a malignant tumor with an extremely poor prognosis. This tumor is highly aggressive and patients with this form of cancer have a short survival after diagnosis. Even when the tumor is localized, the mean survival time after radical resection varies from 10 to 20 months [1]. The mechanisms of the aggressive growth and metastasis are not yet extensively understood.

Several studies indicated that proliferative activity of tumor cells, as well as tumor angiogenesis, inactivation of tumor supressor genes, overexpression of growth factors may play role in pancreatic carcinogenesis and may help to predict patient outcome [2-8]. Recent studies denoted that alterations in growth factors and growth factor receptors seem to influence the biologic behaviour of pancreatic cancer cells [2]. Growth factors are involved in carcinogenesis, where they influence a variety of functions including cell proliferation, invasion, metastasis, angiogenesis, local immune system functions, and extracellular matrix formation [2]. Growth factors do not only stimulate cell proliferation, but they may also act as growth inhibitors, depending on the cell type and the stimulatory pathway that is involved. Transforming growth factor-β [TGF-β] is such an example, being a growth stimulator in fibroblastic cells with TGF-β receptors, but a negative regulator in epithelial cells.

TGF-β belongs to a family of homologous polypeptides that includes three major isoforms [TGF-β1, TGF-β2, TGF-β3]. It has been reported that TGF-β influence different cell functions, including growth, proliferation and differentiation. It can influence cancer growth in various ways, such as by stimulating angiogenesis, suppressing cancer-directed immune functions, increasing the expression of adhesion molecules and extracellular matrix components [9]. Human pancreatic cancer cells may exhibit loss of responsiveness to TGF-β-mediated growth inhibition as a consequence of altered TGF-β expression as well as a result of postreceptor alterations [10]. It has also been demonstrated that TGF-β induced cell cycle arrest can be partially attributed to the regulatory effects of TGF-β on both the expression and activity of cyclin-dependent kinase inhibitors [CDKI] such as p21 and p27. The binding of these inhibitors to spesific cyclin-dependent kinase [CDK] complexes blocks their activity and causes cell cycle arrest [11,12].

Alterations in cell cycle regulatory mechanisms play an important role in the tumor development. Cell cycle progression is regulated by a series of cyclins, CDKs and CDKIs. p27, a member of the Cip/Kip family is a low molecular weight CDKI, which is able to arrest cell cycle progression by complexing CDKs and their activity [13]. Low p27 expression has been reported to be a poor prognostic factor in a variety of human cancers including prostate, lung, squamous cell carcinomas [13-18].

In this study, we investigated the immunohistochemical expressions of TGF-β1 and p27 in pancreatic adenocarcinomas and the results were correlated with the clinicopathologic characteristics of the cases and the patients' survival to find out if these factors could be used as an additional predictor of the disease extent and patient outcome. Additionally, we evaluated the expression of TGF-β1 and p27 in the context of other cell cycle and proliferation markers; cyclin D1 and Ki-67.

## Methods

Pancreatic cancer tissues were obtained from 63 patients [36 female, 27 male] undergoing pancreatic surgery for primary pancreatic adenocarcinoma in Dokuz Eylül University Hospital between the years 1996 and 2003. The mean age of the patients was 62 years, with a range of 42–82. The patients were not subjected to any sort of chemotherapy or radiation therapy prior to resection. Surgical procedures consisted of either distal pancreatectomy or Whipple procedure. Some of the patients underwent palliative surgical treatment. The histological diagnosis of each specimen was provided by standard light microscopic evaluation of the routinely processed and parafin embedded tissues. The H&E stained slides of each case were taken from the pathology archieves and reviewed by two pathologists without knowledge of the patients' outcome. The tumors were typed as proposed by WHO and graded as well, moderate or poorly differentiated. International Union Against Cancer [UICC] TNM classification system [sixth edition] was used for staging. Staging was based on the information at the time of diagnosis. Lymphatic vessel, blood vessel and perineural invasions as well as lymph node, resection margin and adjacent organ involvements were reevaluated in each tumor.

### Immunohistochemistry

The most representative tumor tissue block was chosen from each case and 5 μm sections were taken to poly-L lysin coated slides for immunuhistochemical staining. Standard streptavidin biotin immunperoxidase method was used for immunostaining with TGF-β1 [Santa Cruz, sc-146, California, USA], p27^Kip1 ^[DAKO, Glostrup, Denmark, M7203, dilution: 1/50], cyclin D1 [NeoMarkers, Fremont CA, Clone DCS-6, MS210-P, dilution: 1/50] and Ki-67 [NeoMarkers, Fremont CA, Clone MB67, MS1006-A, dilution: 1/50] antibodies. This indirect immunoperoxidase staining procedure was performed at room temperature. Appropriate tissue sections known to react with both antibodies as positive controls for each primary antibody were also stained simultaneously.

### Analysis of immunohistochemical data

#### TGF-β1 scoring

The stained slides were examined microscopically using the following parameters and semiquantitative criteria. Cytoplasmic immunostaining in tumor cells were accepted as positive. For evaluation of the immunohistochemical results, the intensity of the immunoreaction and the number of the immunoreactive cancer cells were scored on the tissue slides. The intensity of the immunoreaction was classified into 4 levels: 0: no staining, 1: weak staining, 2: moderate staining, 3: intense staining. In addition, the percentage of the immunoreactive cancer cells was recorded; 0, negative; 1, less than 33% positive staining; 2, 33% to 66% positive staining, 3, more than 66% positive staining. A combined score of 0 to 6 was assigned. Tumors given a score 0 to 1 were classified as negative; those given a score of 2 were classified as weakly positive; a score of 3 to 4 was considered moderately positive; and a score of 5 to 6 was considered strongly positive [19].

#### p27 scoring

Approximately 500 cells from the selected area were counted, and the number and percentage of cells with nuclear p27 staining were noted. Cells that had either weak or strong nuclear staining were considered to be positive for p27 protein expression. Cells without nuclear staining were considered negative. The percentage of cells expressing p27 was recorded as the ratio of positive cells-to-the total number of cells counted. p27 expression was grouped as high or low. Patients with low p27 expression had less than 30% of the nuclei in the specimen staining positive, while those with high levels of p27 expression had more than 30% of the nuclei staining for the bound antibody [15].

#### Ki-67 scoring

Fifty-two cases were stained with Ki-67. Nuclear immunostaining in tumor cells were accepted as positive. At least 10 fields containing approximately 500 tumor cells were counted each per case at 400 × magnification. The number of Ki-67-staining reactive cells in each field was determined as a percentage of the total number of tumor cells counted [20].

#### cyclin D1 scoring

Fifty-two cases were stained with cyclin D1. Nuclear immunostaining in tumor cells were evaluated according to Holland et al [21]. Cyclin D1 expression in tumors was graded as negative: less than 10% of cells stained; 1+: 10–50% of cells stained; 2+: 50–75% of cells stained and 3+: more than 75% of cells stained.

### Statistical analysis

Data were analyzed by a computer software SPSS for Windows 10.0. A p value <0.05 was considered statistically significant. Immunohistochemical scores were compared among prognostic parameter subgroups by the chi-square and Fishers exact tests. Correlations between non-parametric and parametric values were tested with Kendall's tau-b and Spearman correlation tests respectively. The effect of prognostic and immunohistochemical scores on the patient survival was tested in potentially curatively operated patients who had R0 resections, with Cox regression analysis.

## Results

### Clinicopathologic features

Clinicopathologic features of the patients is shown in Table 1. Thirty-six were women, 27 were men. The median age of the patients was 62 years, with a range of 42–82. The size of the resected tumors ranged between 1 and 13 cm [median 4.5 cm]. Sixteen tumors were well differentiated, 33 were moderately differentiated, and 14 were poorly differentiated. Four, 23, 3 and 33 patients had stage I, II, III and IV disease. Thirty-nine [61.9%] patients had one and more than one regional lymph node metastasis and 33 [52.4%] patients had distant metastasis. Among resected tumors, 44 [69.8%] showed perineural invasion, 26 [41.3%] showed lymphatic vessel and 21 [33.3%] showed blood vessel invasion. Survival data were available for 54 patients. At follow-up, 38 patients [60.3%] had died of disease. The overall median survival of these 38 patients was 13 months. Sixteen patients were still alive with no evidence of disease. The remaining patients were either lost to follow-up or their death status was unknown. Among the patients with survival data 27 patients had RO resections and these cases were considered in survival analysis.

### Immunohistochemical analysis

**Figure 1:**
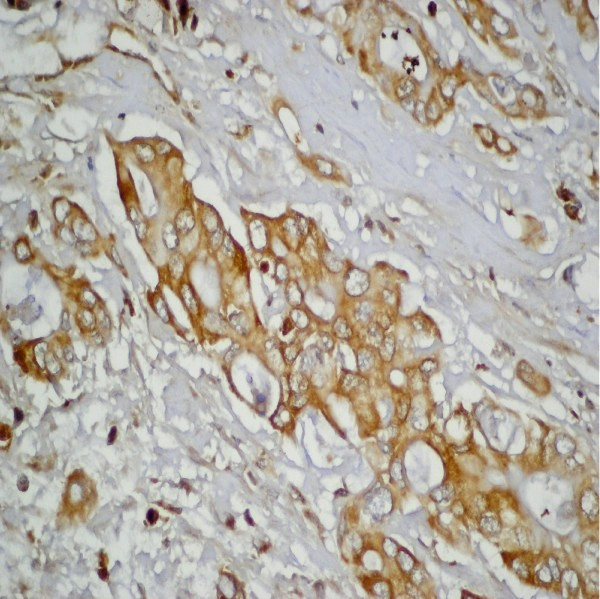
Intense [score 6] cytoplasmic TGF-β1 immunoreactivity of pancreatic ductal carcinoma cells [anti-TGF-β1, original magnification, × 400].

TGF-β1 stained the cytoplasm of the tumor cells in 43 cases [68.3%] [Figure 1]. Strongly positive staining was observed in 14 tissues [22.2%] while 19 tumors [30.1%] were moderately and 10 [15.9%] were weakly positive. There was a statistically significant positive correlation among TGF-β1 staining scores in terms of clinicopathologic factors such as blood vessel invasion [p = 0.01], stage [p = 0.05] and distant metastasis [p < 0.01] [Table 2]. But the other prognostic parameters did not relate significantly with TGF-β1 staining scores. Additionally, we could not find an association with cyclin D1 [Table 3] and Ki-67.

**Figure 2:**
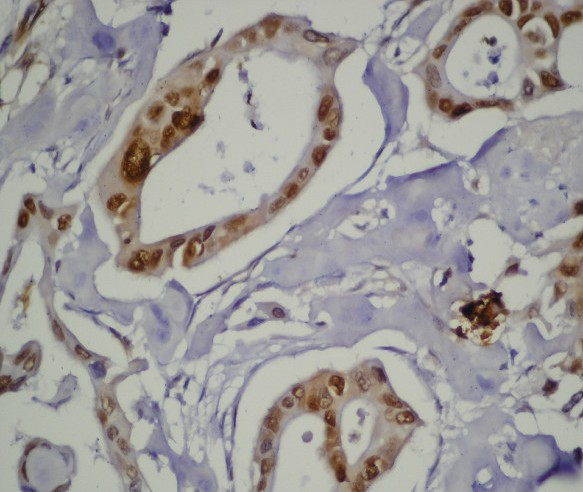
High level nuclear p27 immunoreactivity of pancreatic carcinoma cells [anti-p27, original magnification, × 400].

p27 immunoreactivity was concentrated within the nucleus, but concomitant faint cytoplasmic staining was also observed [Figure 2]. Of the 67 tumors evaluated, 40 [63.5%] were negative, 17 [27%] were assessed to have low grade and 6 [9.5%] were assessed to have as high grade p27 staining. Positive staining for p27 was found dominantly in low-grade tumors [p = 0.02] [Table 2]. Although the number was so small high grade p27 staining tended to be in low stage tumors [stage grouping was performed as: I and II as low, III and IV as high], but the difference was not statistically significant. On the other hand, there was no association between p27 expression and other clinicopathologic parameters. In statistical analysis, there was also no difference between p27 and cyclin D1 expression and Ki-67.

Forty-nine [77.8%] tumors showed positive nuclear staining with cyclin D1 while 3 [4.8%] cases showed no staining. There was a statistically significant difference among cyclin D1 nuclear staining scores in terms of tumor size [p = 0.03]. The other prognostic parameters did not relate significantly with nuclear or cytoplasmic staining with cyclin D1.

In the whole group, the mean proliferative index of the tumors were 41.5%, ranging from 2% to 97%. In statistical analysis, no significant relation was found between mean proliferative index of the tumors and prognostic parameters.

### TGF-β1, p27 and clinical outcome

The median survival time was 13.4 months [range, 0–59 months] for low-expressors and the median survival time was 13.7 months [range, 1–38 months] for high-expressors for TGF-β1. The median survival time was 13.6 months [range, 0–59 months] for low-expressors and the median survival time was 13.4 months [range, 6–26 months] for high-expressors for p27. In the univariate analysis, neither TGF-β1 nor p27 expression was related with patient survival.

## Discussion

Several tumor markers are studied to explain the mechanism of pancreatic carcinogenesis. Dysregulation of the normal cell cycle machinery is integrated to the neoplastic process and there is evidence implicating loss of cell cycle control in the development and progression of most human cancers, including pancreatic cancer. Regulation of cell cycle depends on the complex association between cyclin, CDK and CDKIs. According to their effects on cell cycle progression, these regulators qualify as proto-oncogenes [cyclin D1, D2, E] or tumor supressors [p21, p27]. Recently, studies have been performed in demonstration of p21, p27, cyclin D in tumors [17,22-25].

p27 is a CDKI that negatively regulate cyclin and its low expression results in proliferation of DNA damaged cells. Recent studies indicate that reduced expression of p27 is an independent predictor of poor outcome in cancers such as oral tongue squamous cell carcinoma, prostate cancer, gastrointestinal tract cancers, and even laryngeal precancerous lesions [14-18,26,27]. Lu et al demonstrated that loss of p27 expression independently predicts a poor prognosis for patients with resectable pancreatic adenocarcinomas [24]. Also anomalous overexpression of p27 in human pancreatic endocrine tumors were reported [25]. Inverse correlation between p27 expression and tumor grade, lymph node metastasis and stage of tumor was reported [14]. In the present study, we found positive staining for p27 in only minority of pancreatic adenocarcinoma cases and the staining was weak indicating that p27 is down-regulated during neoplastic process in pancreas. Additionally, although the difference was not statistically significant, p27 was dominantly expressed in low stage tumors. These results confirmed the previous reports. In a few reports, p27 and p21 staining did not correlate significantly with any clinicopathologic parameters [28,29], but there was an association between p27 and cyclin D1 expression indicating that the balance between the two opposing regulators was important for the end result of cell cycle progression [22]. Del Pizzo and Armengol found a positive correlation between p27 and cyclin E [30,31]. In the present study we could not find any relation between p27 and cyclin D1 expression.

It is well established that cell proliferation kinetics are important in predicting the prognosis of various tumors. Recenty, the highly proliferative activity as measured by Ki-67 antigen has been shown to correlate with a reduced p27 expression in various tumors suggesting that decreased expression of p27 may play important role in the increased cell turnover [32]. But the lack of correlation between cell cycle regulators and proliferation index has been also reported in some studies [22,27,31]. In the present study, we did not find any significant association between p27, cyclin D1 and proliferative activity. Cell cycle regulators might be more closely related with differentiation than proliferative activity, so induction of them may perhaps be necessary, but probably not sufficient for inhibition of cell cycle.

Growth factors, such as TGF-β and growth factor receptors have been shown to be overexpressed in a variety of cancers [10,33]. TGF-β can act as both a tumor suppresor and as a stimulator of tumor progression, invasion and metastasis. TGF-β inhibits normal cell proliferation and this effect is induced by binding specific cell receptors, type I [TβR-I_ALK5_] and type II [TβR-II]. Both receptors were present in most cancer tissues and presence of them was associated with advanced tumor stage [10]. In pancreatic cancers, all three TGF-β isoforms are expressed at high levels and the presence of TGF-β isoforms is associated with shorter postoperative survival [34]. TGF-β isoforms were also markedly increased in hepatocellular carcinoma and prostate carcinoma [35-37]. Overproduction of TGF-β1 and loss of TGF-β-RII expression were found to be associated with poor clinical outcome [10]. Nevertheless several reports have indicated that TGF-β1 expression showed a significant correlation with low grade tumors and with longer survival time [19,29]. In the present study there was a statistically significant difference between TGF-β1 staining scores in terms of clinicopathologic factors such as blood vessel invasion, distant metastasis and stage. Our analysis revealed that overproduction of TGF-β1 correlates with tumor progression and metastasis. In the present study we could not find a correlation between TGF-β1 and p27 expression. TGF-β1 might influence cancer growth in other ways rather than cell cycle proteins. Hashimoto demonstrated a significant correlation between TGF-β1 and p21 expression and stated that the evaluation of TGF-β1 and p21 expression might be an effective tool in the prediction of the prognosis of patients with pancreatic cancer [29]. Grau and Ito also reported that TGF-β1 induces p21 expression in pancreatic cancer cells [11,38]. p21 may play more important role in the biology of pancreatic cancer than p27. We found no correlation between TGF-β1 and other proliferation markers studied. TGF-β1 induction of other CDKIs, such as p15 may partially participate in the inhibition of mitosis.

There is conflicting data in the literature about the correlation of TGF-β1 expression with survival in pancreatic cancer. In most series, the presence of TGF-β1 is associated with shorter postoperative survival [34], although in some other studies TGF-β1 expression showed a significant correlation with longer survival time [19,29]. On the other hand, there are reports indicating that TGF-β expression has no association with survival [39]. In the present study, to have the same treatment protocol only the patients which had RO resections were considered in survival analysis and TGF-β1 and p27 expression did not show a significant influence on the survival of patients. The small number of the patients may be the reason not to have definitive conclusions. TGF-β's role in controlling cellular growth and differentiation is very complex. In this study, findings may suggest that TGF-β1 may be a factor in the extention of the disease in pancreatic adenocarcinomas. But, progress may depend on the participation of other regulatory signals. Additional parameters related to prognosis and standard prognostic parameters are needed in the prediction of the disease outcome. Further studies that involve screening a larger number of patients with invasive disease will be required to establish the potential clinical usefulness of cell cyle regulators as prognostic markers in pancreatic cancer.

## Conclusion

In the present study we examined the expression paterns of TGF-β1 and p27, which involved in cell cycle regulation, in pancreatic adenocarcinoma. Our findings suggest that in pancreatic carcinoma, TGF-β1 overexpression is more pronounced in metastatic and progressive ones. p27 expression is reduced in pancreatic adenocarcinomas and it may play a role in the differentiation of pancreatic cancer. Therefore, evaluation of these proteins might be useful in determining the agressive capacity of these tumors at an earlier stage.

## Competing interests

The author(s) declare that they have no competing interests.

## Authors' contributions

NC carried out the pathological studies, drafted and wrote the manuscript

ÖS participited in the pathological studies and performed the statistical analysis

SK, HA, İA performed the surgery and follow-up of the patients as surgeons

MS, İÖ made the endoscopic studies and performed the diagnosis as gastroenterologists

FO made the radiological analysis and participited in the diagnosis as a radiologist

All authors read and approved the final manuscript

## Pre-publication history

The pre-publication history for this paper can be accessed here:



## Figures and Tables

**Table 1:** Clinicopathological features

	***Number of patients [%]***
*Sex*	
Female	36 [57.1]
Male	27 [42.9]

*Histologic grade*	
Well	16 [25.4]
Moderate	33 [52.4]
Poor	14 [22.2]

*Stage*	
I	4 [6.4]
II	23 [36.5]
III	3 [4.8]
IV	33 [52.4]

*Lymph node metastasis*	
Negative	24 [38.1]
Positive	39 [61.9]

*Perineural invasion*	
Negative	19 [30.2]
Positive	44 [69.8]

*Distant metastasis*	
Negative	30 [47.6]
Positive	33 [52.4]

Total	63 [100]

**Table 2:** Tissue marker expressions and tumor characteristics

	TGF-β1					p 27				Cyclin D1		
***Variables***	0	1	2	3	p	0	1	2	p	0	1	p
***Sex***												
Female	13	7	8	8	0.68	26	6	4	0.10	0	31	0.06
Male	7	3	11	6		14	11	2		3	18	
***Grade***												
Well	8	2	5	1	0.58	12	3	1	0.02	2	13	0.42
Moderate	7	6	10	10		23	9	1		1	25	
Poor	5	2	4	3		5	5	4		0	11	
***Stage***												
I	4	0	0	0	0.05	3	1	0	0.97	1	3	0.30
II	11	4	8	0		14	6	3		0	18	
III	0	1	1	1		3	0	0		0	3	
IV	5	5	10	13		20	10	3		2	25	
												
***Lymph node m.***												
Negative	10	6	7	4	0.34	19	7	1	0.34	3	19	0.23
Positive	8	3	6	9		15	7	4		0	22	
***Perineural inv.***												
Negative	3	1	3	2	0.95	6	1	2	0.21	1	7	0.47
Positive	16	8	11	9		28	13	3		2	32	
***Distant met.***												
Negative	15	5	9	1	<0.01	9	4	0	0.51	2	8	0.09
Positive	5	5	10	13		31	13	6		1	41	
***Blood vessel in*.**												
Negative	14	6	10	4	0.01	23	9	2	0.62	3	25	1
Positive	4	3	5	9		13	5	3		1	17	

**Table 3:** Relationships between TGF-β1, p 27 and cyclin D1

	***TGF-β1 immunoreactivity***				
	0	1	2	3	p value
***Cyclin D1***					
0	1	1	0	1	0.6
1	19	6	12	12	
***p 27***					
0	14	6	13	7	0.7
1	5	3	4	5	
2	1	1	2	2	
